# Supplementary material for: Sialyllactose Attenuates Inflammation and Injury of Intestinal Epithelial Cells upon Enterotoxigenic Escherichia coli Infection
Source: Int J Mol Sci. 2025 Apr 18;26(8):3860. doi: 10.3390/ijms26083860 (PMC12027521; doi:10.3390/ijms26083860)
Supplement: Supplementary file 1 [file ijms-26-03860-s001.zip › ijms-3566880-supplementary.pdf]

Supplementary materials

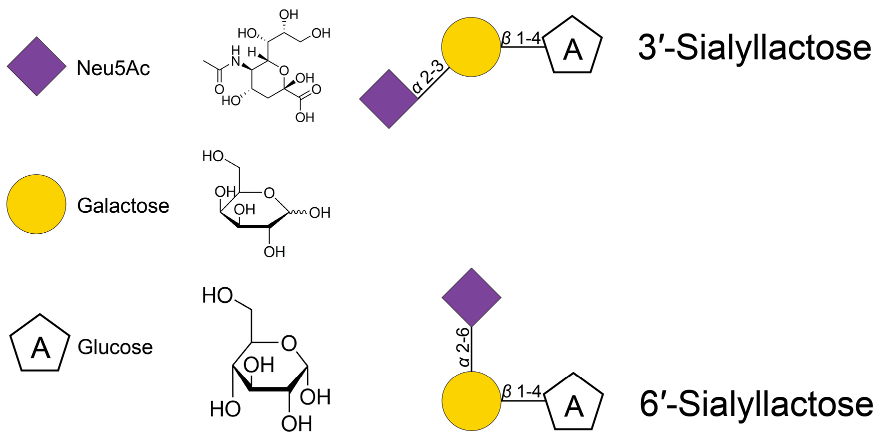

Figure S1. The structures of sialyllactose.

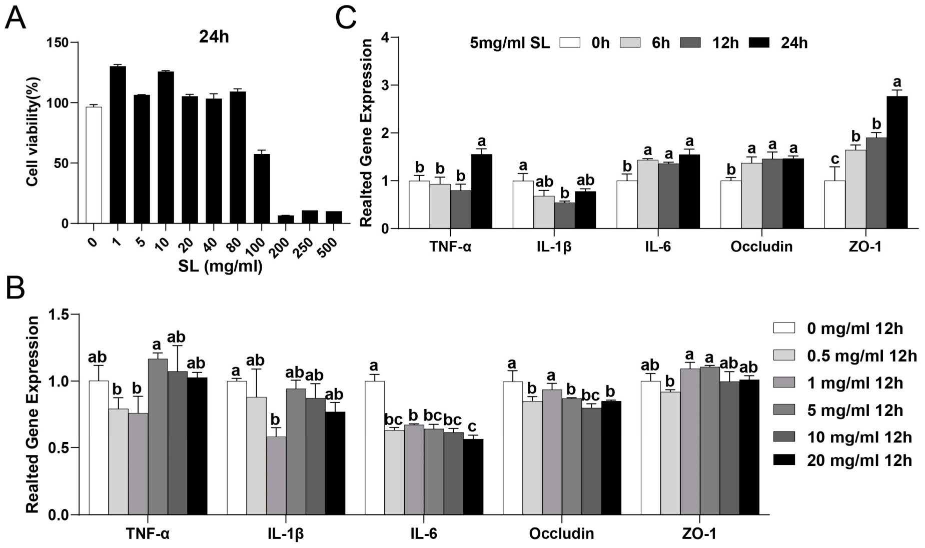

Figure S2. The effects of SL on the cell viability and inflammatory responses of IPEC-J2 cells upon ETEC challenge. (**A**) The IPEC-J2 cells were treated with SL at different concentrations (0–500 mg/mL) for 24 h, then cell activity was determined using Cell Counting Kit-8 (CCK-8). (**B**) The IPEC-J2 cells were treated with SL at different concentrations (0–20 mg/mL) for 12 h, followed by co-treatment with ETEC (1×10^6 CFU) for 1 h, then the expressions of pro-inflammatory and tight junction protein genes were determined by RT-qPCR. (**C**) The IPEC-J2 cells were treated with SL at 5 mg/mL for different amounts of time (0–24 h), followed by co-treatment with ETEC (1×10^6 CFU) for 1 h, then the expressions of pro-inflammatory and tight junction protein genes were determined by RT-qPCR. The data are presented as the mean ± standard error (SEM). The a–c values within each column differ if they do not share a common superscript ($p < 0.05$).
